# Dental Education a Problem

**Published:** 1899-11

**Authors:** Edwin T. Darby

**Affiliations:** Philadelphia


### DENTAL EDUCATION A PROBLEM.

BY EDWIN T. DARBY, D.D.S., PHILADELPHIA.

Mr. President and Gentlemen,—In casting about for a subject upon which to address you this evening, I could think of none more appropriate to a Boston audience than Education, and none more fitting to a dental society than Dental Education. You will observe that I have been announced to speak upon "Dental Education a Problem," which would seem to imply that, in my own mind at least, the best method of dental education has not been fully settled. Although I have been personally identified with college work for a quarter of a century, and have rejoiced at the wonderful progress which has been made during that time, I am not among those who would seem to think that our methods have reached perfection. When dental colleges were established, many of their methods were copied from medical colleges and based upon the assumption that dentistry is a branch of medicine. And so fully has that belief become engrafted in the minds of the followers of the dental profession, that it is quite generally regarded as a specialty of medicine.

It is not my purpose this evening to discuss that phase of the subject. I take it for granted that you are so well satisfied with the character of your calling that you are indifferent as to its place among the professions. You have only to compare its standing to-day with that of fifty years ago, when the first dental college was founded, to assure you that it occupies a position as honorable as any which has for its object the prevention and amelioration of human suffering.

Every profession or calling in life has its successful followers. It has also, alas! its unsuccessful followers. The thoughtful man naturally asks, Why do some men attain eminence in their profession, while other men, seeming to possess equal facilities for preparation, occupy mediocre positions throughout life? Has the fault been in the man, in his training, or in his career after leaving college? Let me put the question in the most practical manner. Why is it that the graduates which the dental schools are annually turning out are so unequal in attainment, so badly matched to run a professional race? Is the man at fault, or has his college training been at fault? And just here we are confronted by our first problem in dental education. Preliminary education undoubtedly has much to do with a student's ability to grasp and retain the teaching of the various branches which are embodied in the dental curriculum, and the well-equipped candidate may be said to possess advantages over the student who has been less fortunate; but this alone does not always account for the difference which is to be found in college graduates.

Let us for the moment assume that each candidate for dental education has graduated from a high school, the requirements of which are equal; and just here let me say, that I trust the day is not far distant when the requirements for entrance to any dental college will be a certificate or diploma from a high school, whose standard shall be as high at least as that required by your Boston schools. When that shall have been attained, then, and not till then, can it be said that the candidate for dental education has received a proper preliminary education. The school with which I am connected is rapidly approaching the time when that will be one of the requirements for admission, and other schools will, I doubt not, as rapidly as practicable, demand the same.

Again, let us assume that our candidate for dental education has a preliminary training fitting him for the special study of any of the liberal professions. Has he the qualifications fitting him for the successful study and practice of dentistry? His preliminary education may fit him for the study of medicine, law, or divinity, for in the prosecution of these the brain and not the hand is the organ to be trained, whereas in dentistry, the hand as well as the head must be educated. The selection of a profession, therefore, is another important problem.

Every-day and commonplace as is this matter of choice, and important as it should be regarded in all its aspects, it nevertheless clearly and conclusively appears, from even the most casual observation, that men often mistake their calling and labor in fields for which they are not fitted, with a hopeless prospect of future success. Could we know the real cause of the many failures in life, I doubt not we should find that the majority of them are due, in part if not wholly, to lack of adaptation, or want of fitness, many having entered professions at the earnest solicitation of parents or friends whose bent or choice would have taken them into the business mart, and others who are wearily handling the yard-stick or posting the ledger would have been in their joyous element pleading to jurors or thundering in the forum. The notion that law, medicine, or some of its specialties, and divinity must be worshipped by the candidate for respectability and honor has done incalculable damage to society. It has spoiled many a good mechanic, many a successful merchant, and has robbed the rural districts of many a good farmer.

But how, you ask, is a young man to know just the niche which nature has intended him to fill in the great cathedral of the world? I am aware that the proclivities of men are not always glaringly manifest in youth, but the bent or tendencies of most men are recognizable at an early age. It would not have required a long argument or intricate process of reasoning to have convinced the young West that he was intended for a painter when he began in the attack and plundered the family cat for bristles to make his brushes; nor Händel, that he was to be a musician when he was frequently stealing interviews with a smuggled clavichord; nor Michael Angelo, when neglecting school to copy drawings, which he dared not bring home; nor Murillo, who filled the margins of his school-books with drawings; nor Pope, who wrote excellent verses at fourteen. In many cases, so early is the preference manifested that it would seem as if the callings, impatient to be chosen, selected their own agents and, storming heart, hands, and brain, made them captive to their will.

It is said "our wishes are presentiments of our capabilities." Can anything be more reasonable than to suppose that he who, attending to the duties of his profession or trade, can gratify the predominant faculty, the reigning passion of the mind, will be the most successful? The very fact that he has an original bias, a fondness or predilection for a certain pursuit, is the best possible guarantee that he will follow it faithfully. His love for it, aside from all other motives, will insure the intensest application as a matter of course. A French writer on agriculture observes that it is impossible profitably to improve land by trying forcibly to change its natural character, as by bringing sand to clay or clay to sand. The only way is to adapt the cultivation to the nature of the soil. So with the moral or intellectual qualities. Exhortation, self-determination, may do much to prick a man on in a wrong career against his natural bent; but when the crisis comes, the artificial character thus laboriously induced will break down, failing at the very time when it is most wanted.

My answer to every young man who asks what pursuit or calling in life he should select would be, Do that which you love most,

—that which attracts you with greatest force. There is hardly a person who is not qualified to shine in some profession or pursuit, and it is better to be at the head of an inglorious calling than at the foot of one which the world calls respectable.

After careful observation extending over a long period of years, I am persuaded that the qualifications essential to the making of a good dentist are much the same as would be required to make a good machinist, a good watch-maker, or a good surgeon, and I am also persuaded that unless a man has these innate qualifications, or can acquire them by dint of perseverance, he will never rank high as a dental practitioner, and until the dental schools of America adopt some plan of ascertaining this peculiar fitness, or the absence of it, they will annually turn out some men of mediocre attainment, who will be handicapped from start to finish. But how to ascertain this peculiar fitness is one of the problems which the colleges have not solved. The responsibility heretofore has been vested in the candidate for admission. It has been presumed that he has weighed the demands which will be made upon him, and has been allowed to enter college and learn for himself his adaptation or the absence of it in this particular line. He has sometimes learned it early in his student life, and has wisely decided upon some other occupation, but too frequently he has persevered in a career for which he knows he is not fitted, and has wasted years of valuable time, only to learn at the close of his college course that he has mistaken his calling. To avoid just such calamities as this should be the aim of the dental schools.

I am aware that it would be difficult to discriminate with any degree of accuracy in every case unless the candidate had been engaged in some pursuit in which manual procedures had been practised. The ideal feeders for dental colleges would be manual training schools, and although it might seem impracticable to insist upon this as one of the conditions of entrance upon a dental course, it would certainly aid materially in determining the fitness of the applicant. Our course in operative and mechanical technics is supplying a long-felt want in this direction. The hand as well as the head is here trained, and the benefits derived from a few weeks in these laboratories is apparent quite early in the student's career.

Again, assuming that our candidate for admission to a dental school has the preliminary education and the manual dexterity, has he sufficient time in the prescribed course to acquire that knowledge which the curriculum imposes? Let us for a moment survey that curriculum. In three years he is expected to acquire a knowledge of anatomy, physiology, chemistry, histology, bacteriology, microscopy, oral surgery, dental pathology, materia medica, therapeutics, mechanical dentistry, metallurgy, operative dentistry, and osteology; and be it observed that in many of these branches he is expected to pass the same examinations as the medical student who sits by his side. Are the colleges demanding too much in the time allowed? Are they demanding too much in the abstract? Is it essential that the dental practitioner should possess a knowledge of the various studies embraced in this curriculum? And if so, ought not some of it to be acquired before entering upon the study of dentistry proper? These are problems which have not been fully solved.

It has been justly said that a great deal of the wisdom of a man in this century is shown in leaving things unknown, and a great deal of his practical sense in leaving things undone. The day of universal scholars is past. "Life is short and art is long." The range of human knowledge has increased so enormously that no brain can grapple with it, and the man who would know one thing well must have the courage to be ignorant of a thousand other things however attractive or inviting. As with knowledge so with work. The man who would get along must single out his specialty, and into that must pour the whole stream of his activities, all the energies of his hand, eye, heart, and brain. Broad culture, many-sidedness, are beautiful things to contemplate, but it is the men of single and intense purpose who steel their souls against all things else that accomplish the hard work of the world. The successful man in any calling is he who can say, This one thing I do. With the exception of a few great creative minds, the men whose names are historic are identified with some one achievement upon which all their life force is spent. You think of Watt, and instantly the steam-engine is suggested; of Arkwright, and the spinning-jenny whirls before you; of Davy, and the safety-lamp lights up the mine; of Harvey, and the blood courses the more quickly in your veins; of Jenner, and you see disease stayed in its progress by the discovery of vaccine; of Morse, and the electric spark is seen darting from continent to continent; of Edison, and the electric light flashes before your eyes. A man may have the most dazzling talents, but if they are scattered upon many objects he will accomplish nothing. Strength is like gunpowder,—to be effective it needs concentration and aim. The marksman who aims at the whole target will seldom hit the bull's-eye.

I am aware that there are exceptions to this rule of exclusiveness or concentration of aim. There have been prodigies of genius who have been able to accomplish many things during an ordinary lifetime, and do these many things well. Thus Cicero was a master of logic, ethics, astronomy, and natural philosophy, besides being well versed in geometry, music, and all the other fine arts. Leonardo da Vinci was not only a great painter, but a mathematician, metaphysician, musician, poet, sculptor, architect, chemist, botanist, anatomist, and astronomer, besides being skilled in mechanics and natural history. The very rarity of such prodigies is what makes them prodigies. To every such instance of universal accomplishment may be opposed thousands who have failed in life by dabbling in too many things. Most men run uncertainly if they have two goals. Vastly better is it to be ignorant of a great many things, and so avoid the calamity of being ignorant of everything.

Do not misunderstand me, gentlemen. I would not seem to speak disparagingly of broad culture or varied accomplishments. The professional man should be a man of culture and refinement. He should be possessed of all knowledge which will bear in the most remote degree upon the specialty which he has chosen. There is no pleasure or satisfaction so great as that which comes through the acquisition of knowledge, and that pleasure is intensified tenfold when one is conscious of acquiring information which will be useful in his daily life; but, unfortunately, to acquire it means time, and time represents capital. In the past our ranks have been filled by young men coming from families in moderate circumstances, and often of inferior preliminary education; but as the entrance requirements have been raised, a better-educated class of men have applied for admittance to our dental schools, and just in proportion to this increasing demand, just in that proportion will better-educated men enter upon the study of dentistry. But the demand for a better preliminary training is not the only one which has been made. The curriculum has also been increased, until to-day it embraces a list of subjects the knowledge of which taxes the student's energies to the fullest extent.

You tell me that the demands made upon the dental student are not greater than those made upon the medical student, but the comparison is not a valid one. Medicine is a science, dentistry is both a science and an art. The skill which the medical man acquires comes after graduation; the skill of the dental student must be shown before graduation. The dental student must not only learn a science, but he must spend months in acquainting himself with manipulative processes and procedures, which in many of the trades require three and four years' time before the apprentice is considered an expert workman. It requires four years to make a good machinist, a good watch-maker, a good jeweller, and yet the dentist is expected to do the delicate work of any of these. To accomplish all that is demanded of the dental student at the present time, and fit him for entrance upon the practice of his profession, another year should be added to the college course.

If unlimited means were at the disposal of those who wish to enter the dental profession, it would be easy to suggest an education which would not only fit the candidate for dental practice, but to occupy a position in society inferior to none. Let me suggest an education which would give the dentist a proper standing in any community. Let his preliminary education be not inferior to that required for a graduation from a high school of superior rank, or, better still, a classical education. Let him spend at least one year in a manual training school, that his hands as well as his head may be trained. Let him graduate from a medical school of recognized worth (not that a medical education is essential to the proper practice of dentistry, but because of the additional satisfaction which such an education would give the candidate, and the confidence which it would inspire in the minds of the public). And then let him spend at least two years in a dental school, from which he could not escape with a diploma until he had shown skill in every detail of practical work.

But you tell me that these are Utopian ideas, and that life is too short to spend so much of it in preparing for a vocation; that the average man would be thirty years of age before he became self-supporting; and the criticism is a just one. The tendency of the age is one of condensation or specialization, and it is to be hoped that the day is not far distant when a University of Medical Science will be established, in which each student may select the studies which bear directly upon the one specialty which he proposes to make his life-work. In such a university many of the studies which are now embraced in the dental curriculum could be taught and the dental course confined more particularly to the teaching of the dental branches.

If the dental schools of America are to continue as at present organized, and the curriculum remain the same, it seems to me that a fourth year should be added to the course, and that year devoted almost exclusively to practice, in both the operative and mechanical departments. It is only by doing over and over again the various procedures that any degree of skill is attained, and it is just this skill which is so essential to qualify the student for entrance upon a dental career. Such additional requirements might deter some from entering upon the study of dentistry, but the profession is not suffering for a large influx. What it needs is better qualified men in its ranks, and the schools which recognize this demand and meet it are the ones which will command the confidence and respect of the dental profession and the world at large.

